# Molecular Pharmacology of Synthetic Cannabinoids: Delineating CB1 Receptor-Mediated Cell Signaling

**DOI:** 10.3390/ijms21176115

**Published:** 2020-08-25

**Authors:** Kenneth B. Walsh, Haley K. Andersen

**Affiliations:** Department of Pharmacology, Physiology & Neuroscience, University of South Carolina, School of Medicine, Columbia, SC 29208, USA; haley.andersen@uscmed.sc.edu

**Keywords:** CB1 receptors, synthetic cannabinoids, molecular pharmacology, cell signaling assays

## Abstract

Synthetic cannabinoids (SCs) are a class of new psychoactive substances (NPSs) that exhibit high affinity binding to the cannabinoid CB1 and CB2 receptors and display a pharmacological profile similar to the phytocannabinoid (-)-*trans*-Δ^9^-tetrahydrocannabinol (THC). SCs are marketed under brand names such as K2 and Spice and are popular drugs of abuse among male teenagers and young adults. Since their introduction in the early 2000s, SCs have grown in number and evolved in structural diversity to evade forensic detection and drug scheduling. In addition to their desirable euphoric and antinociceptive effects, SCs can cause severe toxicity including seizures, respiratory depression, cardiac arrhythmias, stroke and psychosis. Binding of SCs to the CB1 receptor, expressed in the central and peripheral nervous systems, stimulates pertussis toxin-sensitive G proteins (G_i_/G_o_) resulting in the inhibition of adenylyl cyclase, a decreased opening of N-type Ca^2+^ channels and the activation of G protein-gated inward rectifier (GIRK) channels. This combination of signaling effects dampens neuronal activity in both CNS excitatory and inhibitory pathways by decreasing action potential formation and neurotransmitter release. Despite this knowledge, the relationship between the chemical structure of the SCs and their CB1 receptor-mediated molecular actions is not well understood. In addition, the potency and efficacy of newer SC structural groups has not been determined. To address these limitations, various cell-based assay technologies are being utilized to develop structure versus activity relationships (SAR) for the SCs and to explore the effects of these compounds on noncannabinoid receptor targets. This review focuses on describing and evaluating these assays and summarizes our current knowledge of SC molecular pharmacology.

## 1. Introduction

The changing legal and social perception of *Cannabis sativa* highlights the importance of understanding the molecular pharmacology of cannabinoids [1,2,3]. A major breakthrough in cannabis research came with the isolation and identification of (-)-*trans*-Δ^9^-tetrahydrocannabinol (THC) by Gaoni and Mechoulam [4]. THC is the most abundant phytocannabinoid found in *Cannabis sativa* and the main psychotropic compound in the plant [1,2]. The psychoactive effects of THC result primarily from its binding to the cannabinoid CB1 receptor, a member of the G protein-coupled receptor (GPCR) family of proteins [5,6]. CB1 receptors are primarily localized to presynaptic nerve terminals in the central and peripheral nervous system [7,8]. Stimulation of the CB1 receptor causes the dissociation of the βγ subunits of pertussis toxin-sensitive G proteins (G_i_/G_o_) from the α subunit (G_i_α). G_i_α inhibits adenylyl cyclase resulting in a fall in intracellular levels of cAMP [9]. In contrast, G_i_βγ causes the opening of G protein-gated inward rectifier K^+^ (GIRK) channels causing a more negative resting membrane potential [10]. This combination of cannabinoid actions brings about an acute inhibition of synaptic neurotransmitter release [7,11].

Synthetic cannabinoids (SCs) represent a collection of diverse compounds that exhibit high affinity binding to the cannabinoid CB1 and CB2 receptors and display a pharmacological profile similar to that of THC [1,2,3]. A tetrad of behavioral tests has been used to examine cannabinoid-mediated actions through the CB1 receptor in rodents. This cannabinoid tetrad of behavioral responses encompasses hypothermia, catalepsy, antinociception and a suppression of motor activity. WIN 55,212-2, the prototypic aminoalkylindole SC, while displaying equal efficacy to THC shows greater potency in the tetrad tests [12,13]. Other aminoalkylindole compounds, such as JWH-018 and AM-2201, show even greater potency in some of the tetrad paradigms. For example, JWH-018 produces nociceptive activity in the rodent tail flick test with a half-maximal effective dose (ED_50_) of less than 0.1 µmole/kg compared with ED_50_s of approximately 1 and 12 µmole/kg for WIN 55,212-2 and THC, respectively [13,14]. Newer SCs including the indazole carboxamides AB-CHIMINACA and AB-PINACA also produce dose-dependent nociception, ring immobility (catalepsy), hypothermia and suppression of movement consistent with their binding to the CB1 receptor [15].

Products containing SCs, sold under brand names such as K2, Spice and Black Mamba, first became available in the early 2000s and have grown in popularity since that time particularly among teenagers and young adults [1,2]. These products contain a mixture of SCs that are sprayed on dried plant material and marketed to suggest a similarity to marijuana. Alternatively, liquid formulations of SCs can be vaporized and inhaled using e-cigarettes or other vaping devices. While the first K2/Spice products contained the SCs JWH-018 and CP 47,497-C8, manufacturers have introduced newer structural classes of SCs (see below) to avoid forensic detection and drug enforcement scheduling. Adverse pharmacological effects reported from SC use include impairment of fine motor skills, increased blood pressure, tachycardia, tremors, respiratory depression, seizures, ataxia, nausea, vomiting, acute kidney injury and death [1,2]. Of additional concern, SCs typically produce more adverse psychological effects than those experienced with THC including: impairments of attention and concentration, anxiety, panic, agitation, paranoia, hallucinations, violent or aggressive behavior, short-term memory loss and lack of responsiveness [16,17].

This review provides a brief historical overview of SCs and then describes the molecular pharmacology of the compounds. The review focuses on the cell-based assay technologies that have been utilized to reveal CB1 receptor-mediated effects of the SCs and summarizes the results obtained using these technologies. Readers wishing a more in-depth description of the chemical properties of the SCs, or their neurological/clinical effects, are directed to the appropriate reviews [2,16,17,18].

## 2. Overview of Synthetic Cannabinoids

**Figure 1:**
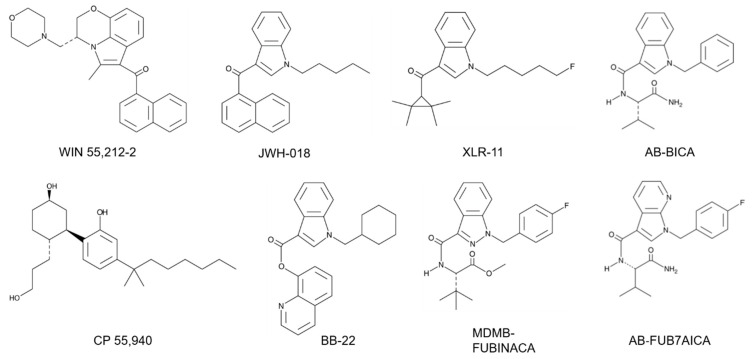
Structural classes of synthetic cannabinoids. Chemical structures of WIN 55,212-2 (aminoalkylindole), JWH-018 (naphtholylindole), XLR-11 (tetramethylcyclopropyl), AB-BICA (indole carboxamide), CP 55,940 (cyclohexylphenol), BB-22 (quinolinyl ester), MDMB-FUBINACA (indazole carboxamide) and AB-FUB7AICA (7-azaindole carboxamide).

Major structural classes of SCs include the naphthoylindoles, phenlacetylindoles, cyclohexylphenols, tetramethylcyclopropyl indoles, indole and indazole carboxamides and quinolinyl esters (Figure 1). The aminoalkylindole class of SCs was developed based on the structure of the nonsteroidal anti-inflammatory drug (NSAID) pravadoline (WIN 48,098) [19].

Structure-activity relationship (SAR) studies carried out by Sterling Winthrop in the late 1980s resulted in the identification of the exemplar CB1/CB2 receptor agonist WIN 55,212-2 [19]. Based on the structure of WIN compounds, John W. Huffman and colleagues at Clemson University synthesized the first series of naphtholylindole cannabinoids (JWH-007, JWH-018, JWH-201, etc.) in the 1990s [13,20,21]. These experiments were designed to develop a SAR for the CB1 receptor and to compare the binding properties of the SCs with THC. This was followed by the synthesis of other naphthoylindoles (AM-1220, AM-2201), naphthoylpyrroles (JWH-30, JWH-145) and phenylacetylindole (JWH-203, JWH-250) compounds [13,22]. Thus, in the early 2000s the aminoalkylindoles along with cyclohexylphenols (CP-47,947, CP-55,940) were the most common SCs found in K2/Spice products.

In the period from 2010 to 2020, new groups of chemically distinct cannabinoids appeared on the SC market. These included the tetramethylcyclopropyl indoles UR-144 and XLR-11 (Figure 1). In these compounds, a tetramethylcyclopropyl group is substituted for the naphthoylindole group found in earlier SCs (e.g., JWH-018). In addition, XLR-11 has a fluoro group (see below) added to the terminal end of the pentyl side chain of UR-144 (Figure 1). Other classes of SCs including indole (AB-PICA, AB-FUBICA) and indazole (AB-PINACA, MDMB-FUBINACA) carboxamides, as well as quinolinyl esters (BB-22, PB-22), were introduced during this time into K2/Spice products. The indole and indazole carboxamides then provided the scaffold for the production of newer chemical moieties [2,3,18]. For example, cumylamine SCs (CUMYL-PICA, CUMYL-FUBICA) are derived through substituting the valinamide (AB) or other amino acid group of the indole and indazole carboxamides with a cumylamine. The azaindole SCs (5F-AB-P7AICA, 5F-PCN) have the indazole group replaced with a two nitrogen atom-containing aziandole (Figure 1). Benzimidazole analogs (FUBIMINA, MEPIRAPIM) replace the indole core with a benzimidazole group. The cumylamine, azaindole and benzimidazole SCs represent three of the newer groups identified by the European Monitoring Centre for Drugs and Drug Addiction (EMCDDA) on the NPS drug market [3].

Stereospecific activity and enhanced cannabinoid receptor affinity with alkyl chain fluorination are two important chemical characteristics of many SCs. Early studies demonstrated that while (+)-WIN 55,212 was active in the mouse tetrad test, its enantiomer (-)-WIN 55,212 lacked activity [12]. HU-210, the (–) enantiomer of 11-hydroxy Δ^8^-THC- dymethylheptyl, is a full agonist at the CB1 receptor [23]. In contrast, the (+) enantiomer (HU-211) has limited cannabinoid activity [24]. Recent studies have also demonstrated stereospecific effects of newer SCs. For some of the carboxamide-type SCs (AB-FUBINACA, AMB-CHMINACA), the (R)-enantiomer shows increased potency at the CB1 receptor compared to the (S)-enantiomer [25,26]. Terminal fluorination of pentylindole SCs is a popular chemical modification found in K2/Spice products. Substitution of a fluorine atom for hydrogen is found in AM-2201 (terminal fluorination of JWH-018) and results in greater CB1 receptor binding affinity [27]. Consistent with this, the fluorinated analogs of the SCs UR-144, PB-22 and APICA (XLR-11, 5F-PB-22 and STS-135, respectively) show increased CB1 receptor potency [28]. Surprisingly, these terminal fluorinated SCs show no greater potency compared with the nonfluorinated compounds when tested in rats for changes in body temperature and heart rates [28].

## 3. CB1 Receptor-Mediated Cell Signaling

**Figure 2:**
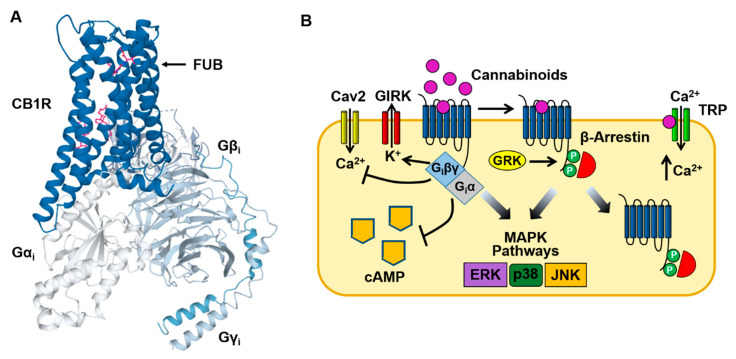
Cannabinoid CB1 receptor structure and signaling. (**A**) Structural model of the CB1 receptor (CB1R)-G_i_ protein complex obtained from cryoelectron microscopy. The binding site for MDMB-FUBINACA (FUB) is indicated by the magenta SC structure. The CB1-G_i_ receptor complex structure was obtained from the Protein Data Bank (code 6N4B). (**B**) Binding of SCs to the CB1 receptor stimulates both neuronal G_i_/G_o_ and β-arrestin signaling pathways (see text for description). In addition, activation of inotropic transient receptor potential (TRP) channels by cannabinoids causes Ca^2+^ influx into the neuron.

Binding of cannabinoids to the CB1 receptor produces a characteristic group of psychotropic effects including euphoria, enhancement of sensory perception, antinociception, appetite stimulation and impairment of memory. As is the case with other Class A GPCRs, the CB1 receptor contains seven transmembrane domains and an intracellular domain that interacts with the G_i_ protein heterotrimer (Figure 2A) [6,29,30]. In the CNS and peripheral nervous systems, the CB1 receptor predominately couples to the G proteins G_i_ ad G_o_ (Figure 2B). However, under some conditions, CB1 receptor-mediated stimulation of G_s_ and G_q_ has also been observed (see Section 5) [31,32]. G_i_ inhibits the production of cAMP and the opening of Ca^2+^ channels (N & P/Q type) while activating GIRK channels [9,10]. These acute actions occur within seconds of cannabinoid binding to the CB1 receptor. This is followed by receptor phosphorylation (by G protein receptor kinase [GRK]) that recruits β-arrestin1 (βarr1) and β-arrestin2 (βarr2) to the receptor and results in CB1 receptor desensitization and internalization [33,34,35]. Both G_i_ and β-arrestin can also stimulate mitogen-activated protein kinases (MAPKs), including the extracellular signal-regulated kinases (ERK1/2), bringing about additional cellular effects [36,37,38]. Finally, cannabinoids can act through membrane receptors (off targets) other than the CB1 and CB2 receptors. Endocannabinoids such as anandamide (N-arachidonoylethanolamine [AEA]) and 2-arachidonoylglycerol (2AG) bind to, and activate, inotropic transient receptor potential (TRP) channels causing cell membrane potential depolarization and Ca^2+^ influx (Figure 2B) [39,40]. In addition, the deorphanized GPCRs GPR55 and GPR18 are also targets of endocannabinoids [41,42].

Accumulating evidence now suggests that SCs function as biased ligands at CB1 receptors. The concept of biased receptor agonism (or functional selectivity) was proposed as a mechanism for explaining how ligands which bind to the same GPCR can produce differing pharmacological actions [43]. It was hypothesized that biased receptor agonists can stabilize GPCR conformations that couple to some signaling pathways, but not to others. The strongest support for the biased agonism model has come from studies with the µ-opioid receptor where ligands that stimulate G protein-dependent pathways have beneficial analgesic actions while ligands that recruit β-arrestins have adverse actions (such as respiratory depression) [44]. Oliceridine (TRV130), a biased µ-opioid receptor agonist, displays an efficacy equal to that of morphine for G_i_ stimulation, but does not promote morphine-mediated β-arrestin recruitment and µ-opioid receptor internalization [45]. Experimental evidence for SC biased signaling is provided in Table 1 and described in Section 5.

## 4. CB1 Receptor Signal Transduction Assays

**Figure 3:**
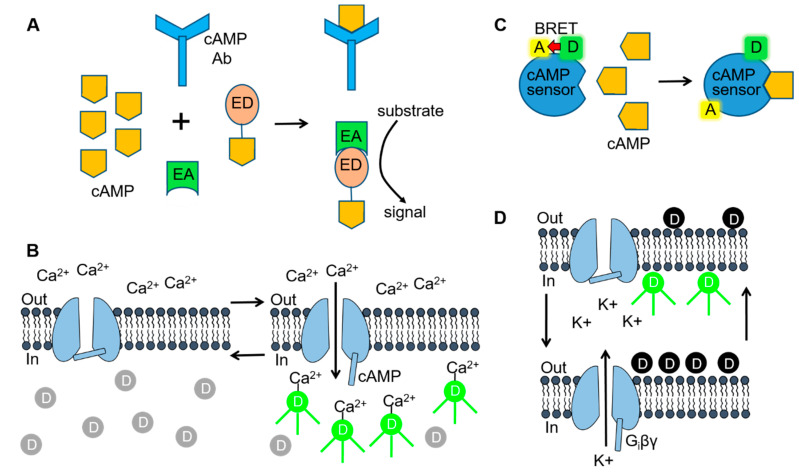
Cell-based assay technologies used for delineating SC-mediated signaling. (**A**) Fragment complementation assays consist of inactive enzyme donor (ED) and enzyme acceptor (EA) components that form an active enzyme when combined. For measuring 3′,5′-adenosine monophosphate (cAMP), the ED consists of an inactive fragment of β-galactosidase that is conjugated to cAMP. When cellular cAMP levels are low or absent, the conjugated cAMP is sequestered by the cAMP antibody and no active enzyme is formed. In the presence of high levels of cAMP (as shown), the ED-cAMP conjugate is free to combine with the EA. β-galactosidase activity is then be detected by adding a substrate that is converted to a fluorescent or luminescent signal. (**B**) Cyclic nucleotide-gated (CNG) channel cAMP assay. Opening of CNG channels during elevations in intracellular cAMP allows Ca^2+^ to enter the cell and bind to Ca^2+^-sensitive fluorescent dye molecules. (**C**) Bioluminescence resonance energy transfer (BRET) assays use a biosensor consisting of a BRET donor (D) and acceptor (A) pair. The BRET cAMP sensor consists of a cAMP binding protein coupled to the BRET donor, Renilla luciferase (RLuc) and acceptor, yellow fluorescent protein (YFP). Binding of cAMP to the sensor (as shown) results in a conformational change and a loss of BRET intensity. (**D**) G protein-gated inward rectifier (GIRK) channel fluorescent membrane potential-sensitive dye (MPSD) assay. Hyperpolarization/depolarization of the cell resting membrane potential (left & right arrows), resulting from G_i_ protein βγ subunit (G_i_βγ) opening/closing of the GIRK channels, alters the distribution of MPSD molecules across the plasma membrane and thus the fluorescent signal. Figure 3A [70] was adapted with permission of Cambridge University Press through PLSclear. Figure 3C,D [71] were reproduced by permission from BMG Labtech and Taylor & Francis Ltd. (www.tandfonline.com), respectively.

While SCs activate a variety of cell signaling pathways, the relationship between the chemical structure of the SCs and their CB1 receptor-mediated molecular actions is only beginning to be explored. In addition, the potency (EC_50_) and efficacy (E_max_) of new structural groups of SCs are not known. As described below, numerous cell-based methodologies are being utilized to study the molecular signaling of the SCs and to establish SAR for SCs on effector pathways (see Figure 3).

### 4.1. GTP Binding

The [^35^S]GTPγS binding assay measures the level of G protein activation following agonist binding to the CB1 receptor. In the assay [^35^S]GTPγS replaces endogenous GTP and binds to the Gα subunit following receptor activation to form a Gα-[^35^S]GTPγS complex. Since the γ-thiophosphate bond is resistant to hydrolysis by the GTPase of Gα, the G protein is prevented from reassembling into the Gαβγ heterotrimer. As a result, the [^35^S]GTPγS-labeled Gα subunits accumulate and can be assayed by measuring the incorporation of [^35^S]GTPγS with a scintillation counter. Since the Gα subunits remain associated with the plasma membrane, cells expressing the CB1 receptor are treated with a SC or control solution, lysed and their membranes collected using filtration. The relative change in the amount of Gα-[^35^S]GTPγS protein, often expressed as a percent increase over basal binding, is then determined by measuring the radioactivity retained on the filter. Although the assay measures the degree of [^35^S]GTPγS binding, it is very useful for quantifying CB1 receptor activation, and thus for determining the potency and efficacy of the SCs [46,47,57,66]. Certainly, the major drawback to this procedure is the inconvenience and cost associated with the handling of radioactive reagents.

### 4.2. cAMP Inhibition

For most cAMP assay protocols, intracellular levels of cAMP are measured in CB1 receptor expressing cells during treatment with the adenylyl cyclase stimulator forskolin (FORS) (with or without the phosphodiesterase inhibitor isobutyl methyl xanthine [IBMX]). These results are then compared with those obtained during the addition of a SC along with FORS and IBMX. Two general methodologies are used in these assays to measure intracellular cAMP. In the first, cAMP levels are measured postexperimentally following cell lysis and the collection of the soluble fraction. One major advantage of these assays is that cAMP can be measured in a variety of cell types and tissue samples. For the most part, these assays are available commercially as kits that use a specific antibody (anti-cAMP Ab) that recognizes both intracellular cAMP and an exogenous cAMP conjugate (Figure 3A). The cellular (or sample cAMP) competes with the cAMP conjugate for binding to the antibody. Detection of the labeled cAMP conjugate is then determined by a variety of methods including enzymatic reactions and Förster resonance energy transfer (FRET). As an example, the HitHunter enzymatic assay (DiscoveryX) uses fragment complementation technology in which one fragment of the β-galactosidase enzyme is conjugated to cAMP (β-gal-cAMP) (Figure 3A) [51,67]. In the presence of high levels of cellular cAMP, the anticAMP Ab becomes saturated, allowing the β-gal-cAMP complex to complement with a second β-galactosidase enzyme fragment and form an active enzyme. The active β-galactosidase enzyme then hydrolyzes a substrate to produce a colorimetric or chemiluminescent signal that is directly proportional to the amount of cellular cAMP.

While commercial kits using a cAMP conjugate provide a reliable and quantitative assay for measuring cAMP, they are relatively expensive, time consuming and involve postexperimental analysis. In contrast, cellular expression of cAMP biosensors provides a more rapid and cost effective approach that measures intracellular cAMP in living cells. One current protocol involves expressing cyclic nucleotide-gated (CNG) channels in cells expressing the CB1 receptor (Figure 3B) [72]. As the name implies CNG channels open during elevations in intracellular cyclic nucleotides [73,74]. CNG channels containing the double mutation C460W and E583M have a high affinity for cAMP but are relatively insensitive to cGMP [73,74]. CNG channels are nonselective cation channels that allow the permeation of Na^+^, K^+^ and Ca^2+^ through the cell membrane. Thus, increases in intracellular cAMP open the C460W/E583M CNG channel allowing Ca^2+^ flux into the cell. The increased Ca^2+^ can then be quantified using a Ca^2+^-sensitive fluorescent dye (e.g., Furo-2, Fluo-4, etc.). Alternatively, cAMP levels are measured in real-time in CB1 receptor cells using a bioluminescence resonance energy transfer (BRET) assay (Figure 3C) [48,50,52]. BRET involves the transfer of energy from a donor luminescence enzyme to an acceptor fluorophore. BRET occurs when the luminescent donor is in close proximity to the acceptor fluorophore. In this protocol, cells are transfected with a cAMP BRET biosensor consisting of a cAMP binding protein (EPAC) coupled to a BRET pair: Renilla luciferase (RLuc) (the donor) and yellow fluorescent protein (YFP) (the acceptor) [75]. Binding of cAMP to EPAC causes a change in the conformation of the protein resulting in a separation of RLuc and YFP [75]. As a result, increases in intracellular cAMP result in a loss of BRET intensity. Finally, GloSensor technology (Promega) uses a mutant form of *Photinus pyralis* luciferase into which a cAMP-binding protein has been inserted [47,49]. Binding of cAMP to the construct causes a conformational change leading to increased luciferase activity and an increased light signal. One major limitation of the cAMP BRET, GloSensor and CNG channel assays is that they require successful cell transfection, making them largely limited for use with heterologous HEK293 and CHO cell lines.

### 4.3. β-Arrestin Recruitment

There are two β-arrestin isoforms, βarr1 and βarr2, found in CB1 receptor-expressing neurons [33,34]. It is speculated that βarr2 induces CB1 receptor desensitization and internalization while βarr1 activates MAPKs (see below) [35]. The PathHunter (DiscoveryX) and NanoBit (Promega) assays have been widely used to study CB1 receptor recruitment of β-arrestins. Similar to the HitHunter cAMP assay, both the PathHunter and NanoBiT technologies use enzyme fragment complementation to measure CB1 receptor-β-arrestin recruitment (Figure 3A) [61,63]. With the PathHunter assay, the CB1 receptor is tagged with one fragment of the β-galactosidase enzyme while βarr2 is tagged with the complementary β-galactosidase fragment. Following binding of the SC to the CB1 receptor and recruitment of βarr2, a fully functional β-galactosidase enzyme is formed. With the NanoBit assay system both βarr2 and the CB1 receptor are fused to an inactive fragment of nanoluciferase termed small BIT and large BIT. In this case, interaction of the complementary nanoluciferase fragments results in a functional enzyme that produces a luminescent signal. One caveat in the use of these technologies is that tagging of βarr2 and the CB1 receptor may affect their trafficking and/or function following heterologous cell expression.

### 4.4. GIRK Channel Activation

Binding of SCs to the CB1 receptor stimulates the dissociation of the βγ subunits of G_i_ from the α subunit. In neuronal tissues, G_i_βγ then activates GIRK (Kir3.1/3.2) channels causing a cellular efflux of K^+^ and a concomitant decrease in the cell resting membrane potential (Figure 2) [10,76]. Therefore, cellular expression of the CB1 receptor and GIRK channels can be used to monitor SC-mediated G_i_ protein stimulation. In these so-called hyperpolarization assays [56,77] cells are loaded with a membrane potential-sensitive dye (MPSD) which distributes across the plasma membrane (Figure 3D). The MPSD molecules inside the cells become strongly fluorescent upon binding to intracellular proteins and other cytoplasmic components. SC activation of the GIRK channels causes the resting membrane potential of the cell to hyperpolarize (more negative potential). As a result, the MPSD molecules redistribute across the plasma membrane with a resulting decrease in the fluorescent signal. New proprietary MPSDs produced by Molecular Devices (FLIPR Membrane Potential Assay kit) and Anaspec (HLB 021-152) provide faster response times and larger fluorescent signals than older oxonol MPSDs. While hyperpolarization assays provide a convenient and inexpensive approach for measuring CB1 receptor-G_i_ signaling, they represent an indirect measure of G_i_ activity when contrasted to the [^35^S]GTPγS binding assay.

### 4.5. MAPK Signaling

Extracellular signal-regulated kinase 1 and 2 (ERK1/2) are serine/threonine protein kinases that serve as essential components of the MAPK signal transduction pathway. CB1 receptor stimulation of G_i_ and β-arrestin results in the phosphorylation of both p42 (pERK2) and p44 (pERK1) (Figure 2) [36]. Traditionally, measurement of p-ERK1/2 was carried out using immunoblot analysis of cell lysates with p-ERK1/2 specific antibodies. The AlphaScreen SureFire p-ERK assay kit (Perkin Elmer & TGR Biosciences) is an immuno-sandwich capture technology that has gained popularity in recent years [78]. The kit utilizes donor and acceptor Alpha beads that are each coated to specifically capture antibodies in the assay. One antibody is specific for the phosphorylation site on p-ERK while the other is a biotinylated antibody specific for another epitope on the protein. In the presence of p-ERK the antibodies bring the donor and acceptor beads in close proximity generating a chemiluminescence signal with an intensity that is proportional to the amount of p-ERK. When compared with immunoblot analysis, one major advantage of the AlphaScreen SureFire assay is that allows p-ERK detection in a multi-well plate format. Thus, this technology can be utilized for high throughput screening of cannabinoids that stimulate ERK1/2.

## 5. Summary of SC Molecular Pharmacology

Table 1 summarizes the effects of SCs on cell signaling pathways with EC_50_s and E_max_s determined using the assays described in Section 4. Like other GPCRs, the CB1 receptor undergoes conformational changes upon ligand binding that are essential for activation of the G_i_ pathway [79,80]. These conformational changes are thought to be facilitated by a conserved network of noncovalent interactions, and that these allosteric rearrangements define activation pathways. Such activation pathways include small groups of adjoining amino acids within the transmembrane domains (TM) that include toggle switches [79,80]. Cryoelectron microscopy (cyro-EM) studies suggest that differences in cannabinoid interaction with the CB1 receptor toggle switch (consisting of residues F200 and W356 in the TM2/TM6 binding pocket) may contribute to different ligand efficacies [80]. For example, strong aromatic interactions of the indazole ring of the SC MDMB-FUBINACA (FUB) with the toggle switch stabilizes the active conformation of the receptor and results in the high efficacy of this ligand (Figure 1; Table 1) [80]. In contrast, the lack of toggle switch interaction may explain why THC acts as a partial agonist at the CB1 receptor [80].

While the results summarized in Table 1 were obtained using various cell systems, experimental methods and transgene constructs, some important generalizations can be drawn from the data. First and foremost, newer indole and indazole carboxylate SCs such as AB-FUBINACA, 5F-MDMB PINACA and 5F-MDMB PICA are far more potent than older SCs including WIN 55,212, JWH-018 and XLR-11. For some of these compounds EC_50_ values measured using GloSensor (cAMP), BRET (cAMP), MPSD (GIRK channel) and NanoBiT (β-arrestin) assays are 100-fold lower than those measured for naphtholylindole and tetramethylcyclopropyl SCs. Secondly, SCs such as JWH-018, 5F-AMB-PINACA and MDMB-FUBINACA display roughly equal potencies in stimulating G_i_ and recruiting βarr2. In contrast, cannabinoids such as CP-55,940 (Table 1), PNR-420 and THC are reported to have less activity in β-arrestin assays when compared with cAMP assays [52,62]. Based on these, and previously reported differences in SC cell signaling profiles, operational models have been applied to calculate ligand bias factors [54,81]. In this analysis, transduction coefficients are calculated from the EC_50_s and E_max_s measured using various signaling assays and compared with a reference compound (such as WIN 55,212-2) [54,81]. Operational analysis eliminates errors that arise from differences in CB1 receptor expression levels, experimental cell type and other confounding factors. As anticipated, bias factor analysis supports the hypothesis that THC and some SCs act preferentially on the G_i_ pathway while other SCs, including indazole carboxylates, are balanced in their signaling actions [52,62].

Not only do SCs differ in their abilities to activate CB1 receptor G_i_ and β-arrestin pathways, but they can also couple to other G proteins within the same cell. While the CB1 receptor predominately couples to G_i_, signaling through G_s_ and G_q_ has also been reported [31,32]. In the presence of pertussis toxin to inhibit G_i_/G_o_, high concentrations of SCs such as WIN 55,212-2, CP-55,940, JWH-018 and AB-FUBINACA increase cAMP levels above those produced by forskolin [48,50]. Interestingly, increases in cAMP are not observed with THC under these conditions, again implying that ligand-receptor interactions modulate G protein coupling [48,50]. In addition to stimulating G_i_ and G_s_, WIN 55,212-2 acts via the CB1 receptor/G_q_/phospholipase C pathway to increase intracellular Ca^2+^ levels in HEK293 cells and cultured hippocampal neurons [32]. Although endocannabinoids such AEA [82] and *N-*arachidonoyldopamine (NADA) [83] are also known to stimulate intracellular Ca^2+^ release, the effect of newer classes of SCs on Gq is currently unknown.

## 6. Conclusions

This review has provided a description of cell-based assays used in the characterization of CB1 receptor-mediated signaling and an overview of SC molecular actions. SCs pose a significant public health risk and represent challenges for hospital ERs because of their wide range of adverse effects. Since 2010 the EMCDDA has reported an alarming increase in the number of SCs on the NPS market [3]. Therefore, it is critical to understand how cannabinoid-CB1 receptor interactions and subsequent cell signaling events bring about both desired and harmful effects. As postulated for µ-opioid receptor agonists, SC-mediated recruitment of βarr1 and βarr2 may contribute to SC toxicity [52,62]. The introduction of new techniques for assessing biased agonism, as well as allosteric and off-target actions of cannabinoids, will be essential in addressing these challenges.

## Figures and Tables

**Table 1:** Summary of the potencies (EC_50_) (in nM) and efficacies (E_max_)^*^ of synthetic cannabinoids (GCs) determined using cell assays.

**Synthetic Cannabinoids**	**GTP Binding**		**cAMP**		**β-arrestin2**		**GIRK Channel**		**Cells**	**Assay**	**Ref.**
**Aminoalkylindoles** **Cyclohexylphenols**	***EC_50_***	***E_max_*** ***(%)***	***EC_50_***	***E_max_*** ***(%)***	***EC_50_***	*E_max_* *(%)*	*EC_50_*	*E_max_* *(%)*			
WIN 55,212-2	100	217							HEK293	[^35^S]GTPγS	[46]
			32	65 ± 5^a^					HEK293	GloSensor	[47]
			13	70 ± 4^b^					HEK293	BRET	[48]
			5	64 ± 6^a^					HEK293	GloSensor	[49]
			46	114 ± 5^c^					HEK293	BRET	[50]
			47	113^c^					HEK293	HitHunter	[51]
			7.6	92 ± 3^c^	1288				HEK293	BRET	[52]
			14	107 ± 1^c^	182	89 ± 2^c^			CHO	Pathfinder	[53]
					570	59 ± 13			Neurons^d^	BRET	[54]
							27	100^e^	AtT20	MPSD	[55]
							282	100^e^	AtT20	MPSD	[28]
							309	100^e^	AtT20	MPSD	[56]
CP-55,940	17	163							HEK293	[^35^S]GTPγS	[57]
	8	199^b^	320	47 ± 8^a^					HEK293	GloSensor	[47]
	0.4	95 ± 3							HEK293	[^35^S]GTPγS	[46]
			0.3	100					CHO	HTRF	[58]
			8	58 ± 3^b^					HEK293	BRET	[48]
			0.6	100	178	57 ± 3^c^			HEK293	BRET	[52]
			0.2	100 ± 3^c^	11	95 ± 2^c^			CHO	Pathfinder	[53]
					138	49 ± 2^c^			HEK293	BRET	[50]
					350	86 ± 4			Neurons^d^	BRET	[54]
							23	100^c^	AtT20	MPSD	[59]
							17	100^c^	AtT20	MPSD	[60]
Naphtholylindoles	EC_50_	*E_max_* *(%)*	*EC_50_*	*E_max_* *(%)*	*EC_50_*	*E_max_* *(%)*	*EC_50_*	*E_max_* *(%)*			
AM-1221	17	163^c^							HEK293	[^35^S]GTPγS	[57]
AM-2201					24	99^f^			HEK293	NanoBiT	[61]
							37	111 ± 6^e^	AtT20	MPSD	[28]
JWH-018	0.7	102 ± 10	1.7	90 ±	78	116 ± 16			CHO	^3^H-cAMP	[62]
					41	99^f^			HEK293	NanoBiT	[61]
			16	64 ± 3					HEK293	BRET	[48]
					37	102^f^			HEK293	NanoBiT	[63]
							18	116^b^	AtT20	MPSD	[28]
JWH-122			3	102^c^					CHO	HTRF	[58]
					72				HEK293	NanoLuc	[64]
N-(5-chloropentyl)					74	289^c^			HEK293	NanoBiT	[61]
N-(5-bromopentyl)					284	261^c^			HEK293	NanoBiT	[61]
N-(5-I=iodopentyl)					215	152^c^			HEK293	NanoBiT	[61]
JWH-210	116	287							HEK293	[^35^S]GTPγS	[57]
					25				HEK293	NanoLuc	[64]
			111	98^c^					CHO	HTRF	[58]
Tetramethylcyclo-propyls	EC_50_	*E_max_* *(%)*	*EC_50_*	*E_max_* *(%)*	*EC_50_*	*E_max_* *(%)*	*EC_50_*	*E_max_* *(%)*			
UR-144					426				HEK293	NanoLuc	[65]
**Synthetic Cannabinoids**	**GTP Binding**		**cAMP**		**β-arrestin2**		**GIRK Channel**		**Cells**	**Assay**	**Ref.**
Tetramethylcyclo-propyls cont.	EC_50_	*E_max_* *(%)*	*EC_50_*	*E_max_* *(%)*	*EC_50_*	*E_max_* *(%)*	*EC_50_*	*E_max_* *(%)*			
UR-144							421	94 ± 4^e^	AtT20	MPSD	[28]
XLR-11							98	110^e^	AtT20	MPSD	[28]
			3981	65 ± 5^a^					HEK293	GloSensor	[47]
			25	107 ± 2^c^	389	88 ± 2^c^			HEK293	BRET	[50]
			63	63 ± 2					HEK293	BRET	[48]
Indole & Indozole Carboxamides	*EC_50_*	*E_max_* *(%)*	*EC_50_*	*E_max_* *(%)*	*EC_50_*	*E_max_*	*EC_50_*	*E_max_* *(%)*			
AB-CHMINACA	7.4	205							HEK293	[^35^S]GTPγS	[66]
			0.28						CHO	HitHunter	[67]
			0.95	100 ± 4^c^	30.9	110 ± 4			HEK293	BRET	[50]
			251	51 ± 2^b^					HEK293	GloSensor	[47]
					3.45	390.5^f^			HEK293	NanoBiT	[63]
							7.8	142^c^	AtT20	MPSD	[60]
ADB-CHMINACA (MAB-CHMINACA)					.34	262.6^f^			HEK293	NanoBiT	[63]
AB-PINACA	71	192							HEK293	[^35^S]GTPγS	[66]
			79.4	69 ± 3^b^					HEK293	GloSensor	[47]
					19	288^e^			HEK293	NanoBit	[61]
							1.2	103 ± 4^e^	AtT20	MPSD	[59]
							6.5	142 ± 15^c^	AtT20	MPSD	[60]
5F-AB-PINACA	2.45	102 ± 7							HEK293	[^35^S]GTPγS	[46]
					66	267^f^			HEK293	NanoBiT	[61]
							0.48	94 ± 6^c^	AtT20	MPSD	[59]
							2.8	132 ± 9^c^	AtT20	MPSD	[60]
5F-ADB-PINACA							0.24	91 ± 7^c^	AtT20	MPSD	[59]
					2.8	308^f^			HEK293	NanoBiT	[63]
ADB-FUBICA							2.6	113 ± 8^c^	AtT20	MPSD	[59]
					12.3	314^f^			HEK293	NanoBiT	[61]
5F-CUMYL-PINACA			0.5	107 ± 6^c^	16.9	97 ± 7			HEK293	BRET	[50]
AB-FUBINACA			0.79	116 ± 4^c^	33	107 ± 4^c^			HEK293	BRET	[50]
			1.36						CHO	^3^H-cAMP	[68]
					16	324^f^			HEK293	NanoBiT	[61]
							1.8	108 ± 7^c^	AtT20	MPSD	[59]
							2.1	151 ± 14^c^	AtT20	MPSD	[60]
ABD-FUBINACA					0.69	339^f^			HEK293	NanoBiT	[61]
							1.20	152 ± 11^c^	AtT20	MPSD	[59]
5F-AMB-PINACA	1.3	96 ± 8^c^							HEK293	[^35^S]GTPγS	[46]
			0.6	98 ± 1^c^	30	100 ± 2			HEK293	BRET	[50]
					15	259^f^			HEK293	NanoBiT	[63]
							1.9	109 ± 3^c^	AtT20	MPSD	[59]
5F-MDMB-PINACA	0.29	111 ± 9^c^							HEK293	[^35^S]GTPγS	[46]
					0.84	319^f^			HEK293	NanoBiT	[63]
					1.78	331^f^			HEK293	NanoBiT	[26]
MDMB-CHMICA			0.14						CHO	HitHunter	[67]
			0.7	117 ± 4^c^	32	108 ± 1			HEK293	BRET	[50]
					1.77	285^f^			HEK293	NanoBiT	[63]
AMB-CHMINACA					3.91	360^f^			HEK293	NanoBiT	[63]
**Synthetic Cannabinoids**	**GTP Binding**		**cAMP**		**β-arrestin2**		**GIRK Channel**		**Cells**	**Assay**	**Ref.**
Indole & Indozole Carboxamides cont.	*EC_50_*	*E_max_* *(%)*	*EC_50_*	*E_max_* *(%)*	*EC_50_*	*E_max_* *(%)*	*EC_50_*	*E_max_* *(%)*			
MDMB-CHMINACA					0.78	227^f^			HEK293	NanoBiT	[63]
MDMB-FUBICA			1.0	108 ± 5^c^	43	104 ± 2			HEK293	BRET	[50]
MDMB-FUBINACA	0.27	75							HEK293	[^35^S]GTPγS	[46]
			0.65	216					CHO	LanceUltra	[69]
					0.36	241^f^			HEK293	NanoBit	[63]
5F-MDMB-PICA			0.17	109 ± 7	20	111 ± 4			HEK293	BRET	[50]
			0.63	60 ± 4^b^					HEK293	BRET	[48]

* - EC_50s_ were normalized as indicated by ^a-f^ (below); a – compared to 65% inhibition with WIN 55,212-2; b – percent of control measurements; c – maximal effect compared to CP55,940 (100%); d – striatal medium spiny projection neurons; e – maximal effect compared to WIN 55,212-2 (100%); f – maximal effect compared to JWH-018 (100%); BRET = bioluminescence resonance energy transfer; MPSD = membrane potential-sensitive dye; HTRF = homogeneous time-resolved fluorescence; NanoBiT = nanobinary technology assay; NanoLuc = nanoluciferase reporter assay.

## Abbreviations

SC	Synthetic cannabinoid
THC	Tetrahydrocannabinol
GIRK	G protein-gated inward rectifier K^+^
SAR	Structure versus activity relationship
GPCR	G protein-coupled receptor
EMCDDA	European Monitoring Centre for Drugs & Drug Addiction
TRP	Transient receptor potential
ERK1/2	Extracellular signal-regulated kinase 1/2
AEA	Anandamide
2AG	2-Arachidonoylglycerol
EC_50_	Half-maximal effective concentration
E_max_	Maximal response
FRET	Förster resonance energy transfer
CNG	Cyclic nucleotide-gated
BRET	Bioluminescence resonance energy transfer
MPSD	Membrane potential-sensitive dye
TM	Transmembrane domains
